# An Experimental Test of the Accumulated Copying Error Model of Cultural Mutation for Acheulean Handaxe Size

**DOI:** 10.1371/journal.pone.0048333

**Published:** 2012-11-08

**Authors:** Marius Kempe, Stephen Lycett, Alex Mesoudi

**Affiliations:** 1 Biological and Experimental Psychology Group, School of Biological and Chemical Sciences, Queen Mary, University of London, London, United Kingdom; 2 Department of Anthropology, Durham University, Durham, United Kingdom; 3 School of Anthropology and Conservation, The University of Kent, Canterbury, Kent, United Kingdom; Institut de Biologia Evolutiva - Universitat Pompeu Fabra, Spain

## Abstract

Archaeologists interested in explaining changes in artifact morphology over long time periods have found it useful to create models in which the only source of change is random and unintentional copying error, or ‘cultural mutation’. These models can be used as null hypotheses against which to detect non-random processes such as cultural selection or biased transmission. One proposed cultural mutation model is the accumulated copying error model, where individuals attempt to copy the size of another individual's artifact exactly but make small random errors due to physiological limits on the accuracy of their perception. Here, we first derive the model within an explicit mathematical framework, generating the predictions that multiple independently-evolving artifact chains should diverge over time such that their between-chain variance increases while the mean artifact size remains constant. We then present the first experimental test of this model in which 200 participants, split into 20 transmission chains, were asked to faithfully copy the size of the previous participant's handaxe image on an iPad. The experimental findings supported the model's prediction that between-chain variance should increase over time and did so in a manner quantitatively in line with the model. However, when the initial size of the image that the participants resized was larger than the size of the image they were copying, subjects tended to increase the size of the image, resulting in the mean size increasing rather than staying constant. This suggests that items of material culture formed by reductive vs. additive processes may mutate differently when individuals attempt to replicate faithfully the size of previously-produced artifacts. Finally, we show that a dataset of 2601 Acheulean handaxes shows less variation than predicted given our empirically measured copying error variance, suggesting that other processes counteracted the variation in handaxe size generated by perceptual cultural mutation.

## Introduction

The idea that human culture – defined here as socially transmitted information such as beliefs, knowledge, skills, artifact designs, and customs – constitutes an evolutionary process was hinted at by Darwin himself in *The Descent of Man*, where he suggested that languages evolve over time in a manner analogous to the diversification and extinction of biological species [1]. This notion of cultural evolution was explored further throughout the twentieth century by archaeologists [2]–[3], anthropologists [4]–[5] and psychologists [6]–[7], but it was not until the work of Cavalli-Sforza & Feldman and Boyd & Richerson in the 1980s [8]–[9] that the implications of the parallels between biological and cultural change were more rigorously explored using the same quantitative mathematical modeling techniques that population geneticists use to successfully model and understand biological evolution (see [10], esp. chap. 3). Our focus here is on the application of these cultural evolutionary methods and concepts to archaeology [11]–[12], which can be seen as the ‘cultural equivalent’ of paleobiology in its aims to document and explain past evolutionary change [13]. This has included the use of phylogenetic methods to reconstruct historical relationships between artifacts [14], the use of models originally developed in population genetics, such as serial founder effect and neutral drift models, to explore the effects of demography on artifact variation [15]–[24], and the explanation of artifact variation in terms of cultural transmission biases such as prestige bias or conformity [21], [25].

Another important process of cultural evolution that may have fruitful application in archaeology is cultural mutation. By analogy to genetic mutation, this describes the process in which ideas are involuntarily changed when they are transmitted from one person to another. In this study we present the first explicit experimental simulation of a model of cultural mutation in archaeology. Specifically, we are interested in testing the accumulated copying error (ACE) model proposed by Eerkens & Lipo [26], in which random error in a quantitative artifact dimension (e.g. size or thickness) is generated by the physiological limitations of the hominin perceptual system. Eerkens & Lipo drew on experimental findings from psychophysics which showed that the accuracy of human perception has physiological limits, especially our ability to perceive differences between objects [27]. If the difference in size between two objects is below some threshold, then this size difference will tend to be imperceptible to the naked human eye, and this will become more and more likely as the size difference between the objects grows smaller. Such error thresholds are always relative to the size of the object, rather than absolute. For example, two lines that are less than 3% different in length are typically perceived as identical, with this 3% value known as the Weber fraction for this particular dimension (line length). Eerkens & Lipo applied this basic principle of psychophysics to the repeated cultural transmission of artifacts. They assumed that when attempting to copy the morphology of an artifact as faithfully as possible, and in the absence of formal measurement aids (e.g. rulers), the manufacturer is likely to make small copying errors that are imperceptible to them due to the aforementioned perception thresholds. If that person's copied artifact is in turn copied by another person, and so on along a transmission chain, then copying errors will compound over time, possibly creating significant morphological change compared to the original artifact. Moreover, if multiple such transmission chains evolve independently, then the variation between these diverging chains is likely to become substantial and to increase over time. Note that this process will take place regardless of whether any other cultural evolutionary forces are at work, and thus, it may be useful to incorporate this model of mutation in other, more complicated models.

Eerkens & Lipo presented a simple simulation model of this process in which a continuous trait value is transmitted over successive generations of individuals with a 3% random normal error rate, and with 10 independently evolving chains. Their simulation showed that, as expected, the independent chains diverged over time as some became larger and others became smaller. Due to the randomness of the error, the overall mean value did not change over time, while the between-chain variation did increase over time. They then applied these expectations to two case studies, showing that the thickness of Owens Valley projectile points increases in variation in a way consistent with the random accumulated copying error model, while the basal width of those points, and the vessel diameter and thickness of Late Woodland pots, show less variation than expected, suggesting that some non-mutation process (e.g. conformist transmission) may have been at work in these latter cases.

Our aim here is to provide an explicit experimental test of Eerkens & Lipo's ACE model of artifact transmission. Although the assumptions of their model are based on previous experimental findings from psychophysics [28], from where their 3% copying error assumption is derived, it is unclear (i) whether this 3% error threshold is uniform across a large population of individuals, or whether there is inter-individual variation in this threshold value (especially given previous findings of substantial individual variability in some perceptual psychometric functions [29]–[30]), and thus how any inter-individual variation affects the robustness of the model; (ii) whether this 3% threshold, originally obtained for simple lines or abstract geometric shapes, also applies to more realistic artifact shapes; and (iii) whether it is valid to simply extrapolate a single individual's perceptual error along successive transmission episodes, or whether there are unexpected dynamics introduced by the compounding of individual errors (Hamilton & Buchanan [31], for example, argued that the compounding of errors causes chains to decrease in size, on average).

To address these issues, we asked multiple chains of participants to copy an artifact image as faithfully as they could, in a direct replication of Eerkens & Lipo's model. In addition, in order to provide an explicit model within which to insert our experimentally-derived copying-error parameter, we also derive two formal mathematical predictions of the model which allow us to test the assumptions of the model with our data. Although this is the first experimental test of a cultural *mutation* model of artifactual evolution, it adds to a handful of other studies that have experimentally simulated cultural transmission dynamics in the archaeological record (e.g. [32]).

Although the findings of our experimental simulation, like Eerkens & Lipo's original model, are in principle applicable to any culturally transmitted artifact, we take a particular interest here in the question of size variation in Acheulean handaxes. Acheulean handaxes were used by various hominin species from at least 1.76 million years ago [33] to at most 0.14–0.12 million years ago [34], and were thus used longer than all other known hominin tools apart from Oldowan artifacts [35]. They were used in Africa, Europe, and Asia, and their temporal span witnessed the evolution of several new hominin species [36]–[37]. Given this extended temporal and geographic spread, it is perhaps unsurprising that patterned variation *within* this technocomplex has been detected in statistical analyses of handaxe shape (e.g. [38]). However, it has also been argued that certain patterns of stability in handaxe form and size (at least within certain bounds) over this temporo-geographic spread might reflect culturally selective constraints for functional or social reasons [39]–[42]. Applying and testing explicit models of evolution by cultural mutation will allow us to investigate the question of handaxe size in a rigorous way, and provide a base for future explicit models of their cultural selection (e.g. for functional or social purposes). As chimpanzee visual acuity is similar to modern human visual acuity [43], it is likely that hominin species would have had similar visual acuity to our modern human participants, and thus that our measured parameters will be similar to those of fossil hominins. Thus, knowledge of the parameters can be used to derive predictions about the amount of variation generated during the temporal span of Acheulean handaxes that we should expect to find in the archaeological record under the ACE model, and thereby connect our microevolutionary experiment to documented macroevolutionary patterns. We therefore use a handaxe image as our ‘experimental artifact’ in the present study, and in the Discussion we ask whether the experimentally-informed ACE model can account for observed patterns of Acheulean evolution.

## Methods

### Model

The ACE model postulates that each chain consists of a number of generations, each of which has one member. In each generation, the sole member copies some continuously-valued attribute of the artifact of the sole member of the previous generation, introducing a randomly determined quantity of copying error. As we expect each member to have a similarly-shaped distribution of copying errors, the Central Limit Theorem justifies modelling the random determination of copying error as drawing a random deviate from some normal distribution. The famous psychophysical finding of Weber's Law, namely, that perceptual errors scale proportionally to the magnitude of the attribute of the object being perceived, rather than being fixed, absolute quantities, justifies multiplying the previous generation's value by the randomly sampled copying error, rather than adding the copying error to the previous generation's value.

**Figure 1:**
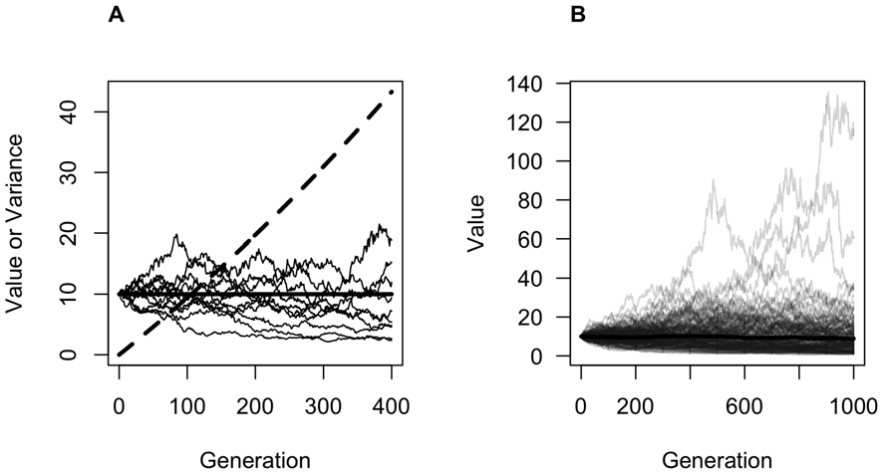
Simulations of the ACE model. (A) 10 chains evolving over 400 generations (black lines) and theoretically predicted mean (thick black line) and variance (thick dashed line). (B) 200 chains evolving over 1000 generations, with individual chains represented by semi-transparent grey lines so that multiple overlapping lines produce darker colors. The thick black line shows the mean of all chains. In both panels, 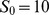 and 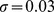.

Thus, we write:
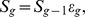
where 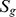 is the value at generation 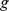, 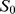 is the starting value of the process, and 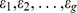 are i.i.d. random variables equal to 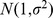. We are interested in the moments of 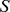, so that we can compare empirical measurements of summary statistics with the model's predictions. Since 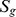 is simply 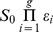, and the error variables are both independent and identically distributed, we can see that:
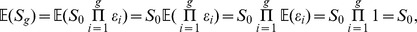
so the expectation of 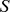 is always equal to its starting value. As for the variance:
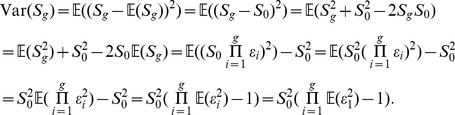



We can find 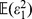 by noting that:
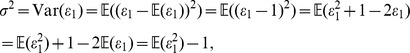
and thus 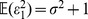, allowing us to find the variance:
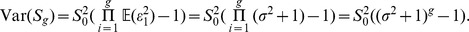



Both of these moments are the moments of a random variable that represents an individual chain, and are therefore unobservable; however, we can estimate them by measuring the mean and sample variance of multiple independently evolving chains, expecting that the mean will stay constant over time and the sample variance will increase without bound. 10 such chains, evolving for 400 generations, are shown in Figure 1A, along with their predicted mean and variance. This partially recreates the results of Eerkens & Lipo [26]. While our analysis confirms that the mean should not change over time, our results suggest that the variance should increase exponentially, rather than plateau. However, when 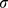 is small (e.g., within the typical range for human copying error distributions) then both our and their equations give very similar predictions for the variance.

We also note that our model and results deviate slightly from a more recent ACE model presented by Hamilton & Buchanan [31]. They found that, in contrast to both Eerkens & Lipo and ourselves, accumulated copying error causes the mean to become smaller. They argued that this is because, given that copying error is relative to the size of the object being transmitted, chains that happen to get smaller will also have smaller copying error, making them less likely to deviate further and more likely to remain small. In contrast, chains that happen to get larger will have larger copying error, increasing the probability that they will eventually produce smaller objects over time. Our results, however, suggests that this is not the case: while it is true that most chains get smaller because small chains stay small, pushing the mean down, this is counterbalanced by a minority of chains that get much larger. Because copying error is relative, those large chains get exponentially large. In other words, small chains stay small, and most chains become small, but large chains get much, much larger, with the overall mean not changing. This can be seen in Figure 1B, which shows the value of most chains drifting smaller than the starting value, a few chains drifting to extremely high values, but the mean of all chains staying basically constant through time. The difference between these results may be due to Hamilton & Buchanan's use of log values, which will reduce the effect of these very large values.

Note that one obvious objection to the above analysis is that normal distributions can take on any value, including negative values, and thus that the resulting values of 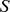 can be negative, which is nonsensical in many interpretations, e.g. if 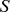 represents size or weight. This is a valid objection in general, but as human perceptual error distributions tend to have very low variance - for example, as we show later, in our data 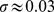 - it makes negligible difference for cultural drift models. For instance, substituting a truncated normal distribution bounded below at 0 with 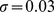 into the equations above gives 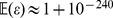, an astronomically small difference that would not affect predicted means and variances even after millions of generations.

### Experiment

**Figure 2:**
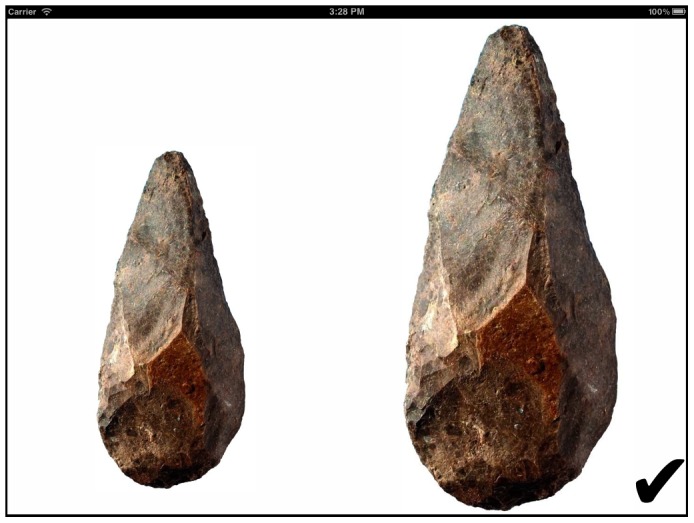
The main screen of the iPad-based experiment. The handaxe image on the left was created by the previous participant, and the current participant is asked to resize the handaxe image on the right so as to match the size of the previous participant's as closely as possible. Participants pressed the tick mark to complete the experiment.

In our experiment, we wish to (1) estimate 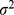, the variance of the distribution of copying errors, and (2) test whether the mean and sample variance of multiple independently evolving chains in an experimental setting match their expected values. Ideally, we would do this by running multiple transmission chains in which participants would be asked to create a new Acheulean handaxe by faithfully copying the previous participant's handaxe. However, Acheulean stone knapping is both dangerous and difficult [44]–[45], and finding enough participants who would be both willing and able to knap handaxes would be a challenge. Thus, we settled on a compromise that allowed us to simulate the essential features of the model: an electronic, touch-screen-based resizing task. Using an iPad, each participant in each transmission chain was shown the previous participant's handaxe and asked to resize a second handaxe to match the size of the previous participant's as closely as possible (Figure 2). This resizing was done using a pinching gesture with two fingers on the iPad screen, and as much time was given as needed; thus, we feel justified in assuming that manufacturing error, as opposed to perceptual error, was not a significant factor in the results of the experiment. It should be emphasized that our transmission-chain experiment thus focuses solely on the ability of participants to replicate the attribute of artifact *size*, to the exclusion of shape attributes. A demonstration of one round of the experiment is given in the movie in the supplemental materials (Video S1).

In our experiment, then, the continuous value modelled as 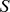 in our model is the size of the handaxe, with height and width scaled isometrically. As the right-hand handaxe image (the one that is to be resized by the participant) must begin at some arbitrary size, we ran two conditions of the experiment: one in which the right-hand image began at the maximum possible size (i.e. with the same height as the screen, 14.4 cm), and one in which it began at 1/3 the size of the screen (4.8 cm height). The zeroth-generation left-hand side handaxe image in each transmission chain was set at 10 cm height (i.e., 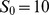), and the width of all images was always 7/15 of their height.

We ran 10 transmission chains with 10 participants each in both conditions. All participants were distinct, i.e., no participant took part in more than one chain or more than once within a chain. Participants were recruited primarily by soliciting in the library of Queen Mary, University of London. 59.5% were female and 75.5% were within 18 and 25 years of age. Those participants who wore corrective eyeglasses or contact lenses were allowed to keep them on for the experiment.

### Ethics statement

The study was approved by the Queen Mary Research Ethics Committee. All participants viewed an informed consent screen and agreed to it by tapping an electronic button; this procedure was approved by the Research Ethics Committee. All data was analyzed anonymously, and gender and age information was deleted after calculating summary statistics across the whole sample.

## Results

**Figure 3:**
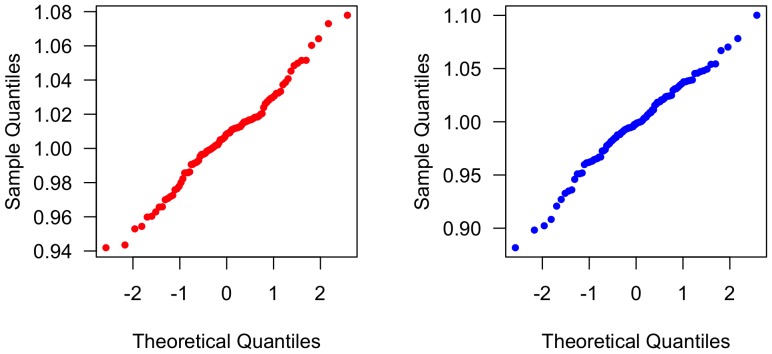
Normal probability plots of empirically measured copying errors. Data from the condition with the larger initial size of handaxe image is red and from the smaller condition in blue.

Our full results dataset is available in the supplemental materials (Data S1). Our first aim was to estimate 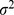. Figure 3 shows normal probability plots (in which a straight diagonal line at y = x indicates perfect fit to a normal distribution) for the distribution of empirically measured copying errors in each condition. For each transmission event, copying error is measured by the final size of the right hand image divided by the size of the left hand image. As can be seen, they appear normal; in order to formally test this hypothesis, we used the Anderson-Darling normality test, which did not reject normality for either distribution (larger condition: A = 0.53; p = 0.17; smaller condition: A = 0.44; p = 0.29). Having established their normality, we can estimate 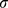 by measuring the sample standard deviation (we report the sample standard deviation here rather than the sample variance to avoid reporting very small numbers, and also because standard deviations are easier to interpret, being measured in physical units rather than units squared), which was 0.0269 for the larger condition and 0.0399 for the smaller condition, with an overall mean of 0.0343.

**Figure 4:**
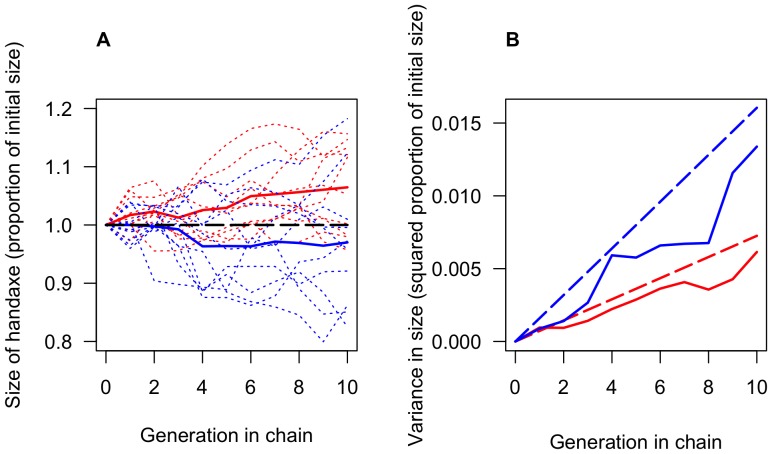
Results of the experiment compared to theoretical predictions. (A) Empirically measured sizes in each chain (thin dotted lines) and means across all chains in each condition (heavy solid lines) in both conditions. Data from the larger condition is plotted in red and data from the smaller in blue. The dashed black line shows the theoretically predicted mean. (B) Empirically measured variances across all chains in each condition (solid lines) and theoretically predicted variances (dashed lines) derived by using the empirically measured variance of the copying error distribution in each condition. Data and predictions from the larger condition are plotted in red and from the smaller condition in blue.

Our second aim was to test the two predictions of the model. Figure 4 shows the empirically measured sizes, means, and variances of the chains over time, and their fit to the predicted values calculated according to the equations derived above. As 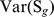 depends on 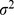, the empirically measured values of 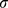 for each condition were substituted into the expression in order to calculate the predicted variances plotted in Figure 4B. As can be seen, the measured means do not seem to fit the predicted mean well, but the measured variances do seem to fit the predicted variances. In order to formally test these hypotheses, we simulated the process described by the theoretical model, substituting in the empirically measured variances for each condition's distribution of copying errors, and matching the conditions of our experiment (i.e. 10 chains of 10 generations each in each condition). This was done with R [46] using code given in the supplemental materials (Code S1). We derived empirical p-values by measuring the proportion of times that a value equal to or more extreme, in the appropriate direction, than the measured final mean and variance in each condition occurred over 10,000 simulations. For the larger condition, the proportion of simulations where the final mean was equal to or more extreme than the empirically measured final mean was 0.01, and the proportion where the final variance was equal to or more extreme than the measured final variance was 0.44; for the smaller condition, 0.22 and 0.42. Thus, our visual intuitions are partly vindicated: the final mean in the larger condition does deviate from the predicted mean more than expected by chance at the 5% significance level, but the final mean in the smaller condition does not, while the final variances in both conditions do indeed not deviate from the predicted variances more than expected by chance at this significance level.

## Discussion

The aim of this study was to provide the first explicit experimental test of the accumulated copying error model of cultural transmission, in which artifact variation increases due to imperceptible differences between a copy of an artifact and the original copied artifact. Acheulean handaxe images were transmitted along 20 independent chains each containing 10 participants, allowing us to measure inter-individual variation in copying error (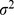) which has previously only been assumed from the psychophysics literature, in which transmission error and artifact evolution are not the focus of study. We find that the ACE model gives good predictions of between-chain variance over time (see Figure 4B): in both the model and the experiment, between-chain variation increases exponentially over time as copying error causes different chains to diverge. Moreover, the empirically determined estimate of 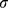 of 0.0343 resembles quite closely the copying error assumed in previous models of 3% [26] which was derived from the psychophysics literature. This supports the use of this assumption in a cultural transmission context.

However, the empirical between-chain mean did not follow the predicted mean in the ‘larger’ condition, in which the initial size of the participants' handaxe was larger than the target handaxe. It is also suggestive that in the ‘smaller’ condition, in which the participants' handaxe started smaller, the measured between-chain means trended below the predicted mean, although the difference between the measured final mean and simulated final means was not significant at the 5% level. It will require more experimental testing to establish whether these biasing effects of the initial size of the object to be resized on its final size are not an artifact of using an iPad. If they are valid effects, they will have interesting implications for predicting ACE in archaeological data, as we would be led to expect that the size of artifacts created by ‘additive’ production methods (e.g. the weaving of baskets) as opposed to ‘reductive’ production processes (e.g. the manufacture of flaked stone tools) would evolve differently, with the size of additively-produced artifacts decreasing slowly through time and the size of the reductively-produced artifacts increasing, at least in instances where there is an effort to replicate faithfully the size of previously produced objects.

As the experiment shows that the model gives good predictions of between-chain variance, and we have estimated the shape parameter of the distribution of copying errors, we are now able to examine whether the model explains known data about the evolution of Acheulean handaxes. Happily, there exists a large database of morphological measurements on Acheulean handaxes, the Acheulean Biface Database [47], against which we can test the model. The database includes length and breadth measurements for 2601 complete handaxes from 21 different sites in 5 countries (Morocco, South Africa, Tanzania, Israel, United Kingdom), with an age range of 1.5–0.3 million years ago. The coefficients of variation for length and breadth in this sample are 0.30 and 0.23, respectively. As deriving an expression for the coefficient of variation of all the artifacts created by a large number of independent chains over time is analytically difficult, we used simulations to estimate this quantity. The simulations were programmed in R using the general form *cv (c (replicate (100, cumprod (rnorm* (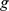, *1*, 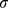 *)))))*. Setting 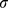 to our measured value of 0.0343, we find that the ACE model will generate 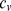 values greater than 0.30 in less than 200 generations, implying an obviously unrealistic lifespan of 4000 years for Acheulean handaxes (assuming a generation time of 20 years). Alternatively, we can set 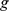 to 60,000, corresponding to 1.2 million years of evolution, the age range of the dataset, if each generation lasts 20 years, which shows that 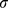 must be approximately 0.0017, or 20 times smaller than our measured value, in order to generate the measured 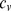 values. Since some of our participants wore eyeglasses, our measured value of 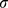 probably errs towards being smaller than a typical ancient hominin value, which emphasizes the mismatch between our model and the data even further. Thus, as a general phenomenon, it is extremely unlikely that Acheulean handaxe size drifted as described by the ACE model.

Before fully accepting this conclusion, however, we should note some limitations of our analysis. First, the ACE model is potentially simplistic in its assumption that all of Acheulean evolution took place in independent lineages; incorporating empirical data on the amount of branching that occurred into the model may allow it to make more realistic predictions. Second, although large, the comparative handaxe dataset used here is not exhaustive in terms of regional or temporal coverage and provides only a broad guide to how Acheulean handaxe size variation compares to the ACE model. While our data suggest that at its broadest scale Acheulean handaxe size variation does not conform to the ACE model, this does not rule out more localized instances of such drift. Indeed, regionally-specific trends of temporal change in handaxe size have been suggested previously (e.g. [48]–[50]), including geographically-localised instances of cultural drift that represent deviations from wider patterns due to situationally-specific circumstances (e.g. in India [51]). Recent analyses have emphasized how spatial and temporal factors might affect cultural patterning under neutral conditions (e.g. [16], [31], [52]–[53]). Given these factors, an important future extension of this study may therefore be to incorporate more explicit geographical parameters into the copying error model (e.g. spatial factors) and compare these revised models against artefactual data with high temporal and spatial resolution.

Assuming that Acheulean handaxe size *does* broadly deviate from the ACE model, we see three possible explanations for this deviation. Firstly, concepts of appropriate limits for handaxe size may have been stabilised by functionally-related cultural selection: for example, by the need to fit into tool users' hands, a highly plausible selective pressure [54], [41]. Secondly, handaxe size may have drifted in a way that stabilized variation: some models of this for quantitative traits were given by Cavalli-Sforza and Feldman [8]. A third explanation for the suggested deviation from the ACE model might be due to the possibility that firm concepts of handaxe *size* (opposed to handaxe production methods leading to their essential and distinctive *shape* properties) may not strictly have been socially transmitted at all. An alternative possibility here is that as functional handheld tools, individuals gained an intuitive sense of what a ‘good sized’ handaxe was via their own empirical engagement with material properties and their various outcomes during usage. This idea resembles a hypothesis proposed by Tennie & Hedwig [55], who noted that some traits in great ape cultural traditions might have been fostered by stimulus enhancement of the trait's raw materials. This may also mean that (somewhat like shoes or other items of clothing) what is an ‘optimally-sized’ handaxe may vary somewhat from individual to individual depending on their own physical size, strength, etc., in turn leading to patterns of variation in handaxe size that deviate from the ACE model. We note, however, that within any socially-mediated context of observation and learning about handaxe production and usage, some notion of suitable size parameters is also likely to have been inducted in novice handaxe producers. Of course, some combination of these causes is also possible. Each of these explanations suggests a number of promising directions for further research.

In conclusion, we have provided a theoretical reformulation and novel experimental test of the ACE model of cultural mutation, in which artifacts change purely due to imperceptible differences between a copied artifact and the original, and which has been proposed as a null model for the cultural evolution of artifacts in the material record. Our experimental test supports the prediction that ACE causes artifact size variation to increase exponentially. However, it did not fully support the prediction that mean artifact size should remain unchanged, instead finding that the initial size of the to-be-copied artifact may bias the eventual copied artifact size. This suggests that the ACE model needs to be revised to incorporate this priming or biasing effect, and that future empirical work might seek to test this effect by comparing reductive and additive technologies. Finally, having established experimentally the validity of the ACE prediction concerning artifact size variation, we apply this prediction to an actual empirical dataset, showing that Acheulean handaxes do not fit the expectation of the ACE model, and we suggest potential alternative explanations for this deviation.

## Supporting Information

Video S1 **A demonstration of one round of the experiment.**
(MOV)

Data S1 **Experimental data.**
(CSV)

Code S1 **R source code for statistical simulation.**
(R) Click here for additional data file.
